# Classification and visual explanation for COVID-19 pneumonia from CT images using triple learning

**DOI:** 10.1038/s41598-022-24936-6

**Published:** 2022-12-02

**Authors:** Sota Kato, Masahiro Oda, Kensaku Mori, Akinobu Shimizu, Yoshito Otake, Masahiro Hashimoto, Toshiaki Akashi, Kazuhiro Hotta

**Affiliations:** 1 Department of Electrical, Information, Materials and Materials Engineering, Graduate School of Science and Engineering, Meijo University, Shiogamaguchi, Tempaku-ku, Nagoya, Aichi 468-8502 Japan; 2 Information Strategy Office, Information and Communications, Nagoya University, Nagoya, Aichi Japan; 3 Graduate School of Informatics, Nagoya University, Nagoya, Aichi Japan; 4 Institute of Engineering, Tokyo University of Agriculture and Technology, Koganei, Tokyo, Japan; 5 Graduate School of Science and Technology, Nara Institute of Science and Technology, Nara, Japan; 6 Research Center for Medical Bigdata, National Institute of Informatics, Tokyo, Japan; 7 Department of Radiology, Keio University School of Medicine, Tokyo, Japan; 8 Department of Radiology, Juntendo University, Tokyo, Japan; 9 Department of Electrical and Electronic Engineering, Faculty of Engineering, Meijo University, Nagoya, Aichi Japan

**Keywords:** Biomedical engineering, Computer science

## Abstract

This study presents a novel framework for classifying and visualizing pneumonia induced by COVID-19 from CT images. Although many image classification methods using deep learning have been proposed, in the case of medical image fields, standard classification methods are unable to be used in some cases because the medical images that belong to the same category vary depending on the progression of the symptoms and the size of the inflamed area. In addition, it is essential that the models used be transparent and explainable, allowing health care providers to trust the models and avoid mistakes. In this study, we propose a classification method using contrastive learning and an attention mechanism. Contrastive learning is able to close the distance for images of the same category and generate a better feature space for classification. An attention mechanism is able to emphasize an important area in the image and visualize the location related to classification. Through experiments conducted on two-types of classification using a three-fold cross validation, we confirmed that the classification accuracy was significantly improved; in addition, a detailed visual explanation was achieved comparison with conventional methods.

## Introduction

The outbreak of the coronavirus disease-2019 (COVID-19) has spread throughout the world, and the number of infected people continues to increase. A method called a reverse transcriptase polymerase chain reaction (RT-PCR) is used to test for COVID-19 infection; however, its accuracy varies from 42 to 71% and it takes longer to receive the test results than other methods^1^. Because the number of infected individuals is expected to increase in the future, the establishment of a highly accurate test method is required. In this study, we aim to establish an automatic classification method of pneumonia incurred through COVID-19 from CT images of the lungs using deep learning. In recent years, studies on the automation of image diagnosis using deep learning have been actively conducted in the medical field^2–17^, and it is known that a diagnosis using deep learning can provide highly accurate and objective results. If a direct diagnosis from CT images can be made possible, the number of people involved in the RT-PCR and the risk of infection will be reduced. A reduction of the inspection time and an increase in the number of inspections will be also expected.

Based on this same idea, many classification methods for COVID-19 using deep learning have been proposed^2–11^. However, with these conventional methods, two important problems have yet to be solved: (1) Although there are differences in CT images of the lung for pneumonia caused by COVID-19 and pneumonia caused by other diseases, such differences vary depending on the progression of the symptoms and the location of the infected area. (2) Most conventional methods aim to obtain a high accuracy and have difficulty finely visualizing the location related to the classification. Problem (1) indicates that the datasets will contain a variety of images, and we consider conventional training methods to be insufficient to acquire an effective feature representation for classification. Problem (2) indicates that conventional methods for a visual explanation are unable to provide a detailed interpretation because the visualization result is based on compressed and high-dimensional information from the network.

To solve these problems, we present a novel classification method based on three types of learning, i.e., classification learning, contrastive learning, and semantic segmentation. Contrastive learning is able to close the distance of image features in the same category and create a better feature space for classification. With the proposed method, we apply supervised contrastive learning^18^. By concurrently applying two different types of training, the classification accuracy is improved based on the differences between images. In addition, we adopt a pixel-wise attention module in the above method. This module is composed of a semantic segmentation, and is able to emphasize an important area in an image and visualize the location related to classification.

We evaluated our method on a dataset of CT images of COVID-19 patients. Based on the experiment results, we confirmed that the proposed method achieves a significant improvement in comparison with conventional classification methods for COVID-19^4,7^.

This paper is organized as follows. We describe related works, the details of the proposed method, and the experiment results. Finally, we summarize our approach and describes areas of future study.

Our contributions are as follows:

- The proposed method trains both classification and contrastive learning at the same time, and generates a better feature space for classification even if the dataset contains images under different conditions.
- Furthermore, in the classification model, we adopt an attention mechanism based on semantic information. It teaches an important location for COVID-19 infection to the classifier and provides a high accuracy and easy-to-understand visual explanation.
- Unlike conventional contrastive learning^18–22^ and other visualization methods^23–28^, our proposed method does not require two-stage learning. It is possible to create a classification and visual explanation using a single model.

## Related works

In recent studies, COVID-19 infection classification from diagnostic imaging has been frequently achieved using a convolutional neural network (CNN)^2–11^. Li et al.^2^ proposed a three-dimensional CNN for the detection of COVID-19. This approach is able to extract both two-dimensional local and three-dimensional global representative features. Wu et al.^3^ proposed a multi-view fusion model for screening patients with COVID-19 using CT images with the maximum lung regions shown in axial, coronal, and sagittal views. In recent years, a new network architecture called a vision transformer revolutionized image recognition and was also used for COVID-19 infection classification. Cao et al.^10^ converted three-dimensional datasets into small patch images and applied them to a vision transformer (ViT). In addition, Hsu et al.^11^ proposed a convolutional CT scan-aware transformer for three-dimensional CT-image datasets used to fully discover the context of the slices. They extracted the frame-level features from each CT slice, followed by feeding the features to a within-slice-transformer to discover the context information in the pixel dimensions.

Although various classification methods have been proposed, there are few methods specializing in visual explanations for COVID-19. A visual explanation enables humans to understand the decision making of deep convolutional neural networks, and it is important to elucidate the cause of this disease in the medical field. Our method is able to classify pneumonia from COVID-19 and visualize an abnormal area at the same time.

### Metric learning

Metric learning can create a space in which image features within the same class are closer together and images of different classes are kept at a distance. It is known to be highly accurate in various tasks such as face recognition^29–33^, object tracking^34–39^, and anomaly detection^40,41^. Contrastive learning, which is a type of metric learning, has attracted attention as a self-supervised learning for obtaining a better feature space^18–22^. Chen et al.^19^ proposed a simple framework for contrastive learning of visual representations, called *SimCLR*. They indicated that data augmentation plays a critical role in defining effective classification tasks, and introducing a learnable nonlinear transformation between the representation and the contrastive loss substantially improves the quality of the representation. In addition, Khosl et al.^18^ proposed supervised contrastive learning that extends the self-supervised contrastive approach^19^ to a fully supervised setting, allowing us to effectively leverage label information. Contrastive learning is also used by certain tasks for COVID-19 screening^12–14^.

Although these methods achieved a high performance for image representation learning, most of contrastive learning consists of two learning stages, i.e., feature extraction and classification. This leads to complicated training and require a lengthy amount of time. Following this problem, Wang et al.^42^ proposed a hybrid framework to jointly learn features and classifiers, and empirically demonstrated the advantage of their joint learning mode. A good point of this method is the reduced training time and more effective features acquired by training through both classification and contrastive learning at the same time. We adopt this idea and achieve to generate a better feature space even if there are various types of images under different conditions in the dataset.

### Visual explanations from convolutional neural network

Several visual explanation methods, which highlight the attention location, have been proposed for convolutional neural networks. The most typical methods are based on a class activation map (CAM)^23–28,43–45^. A CAM can visualize an attention map for each class using the response of a convolution layer and the weight at the last fully connected layer. Because attention maps are represented by a heat map, they are easy for humans to understand. Selvaraju et al.^23^ proposed gradient-weighted class activation mapping (Grad-CAM), which is a type of gradient-based visual explanation. Grad-CAM visualizes an attention map using positive gradients at a specific class during back propagation, and has been widely used because it can interpret various pre-trained models using the attention map of a specific class. In addition, Fukui et al.^44^ also applied a CAM to an attention module called an attention branch network (ABN). An ABN is able to simultaneously train for a visual explanation and improve the performance of the image recognition in an end-to-end manner. Our visualization method is inspired by an ABN.

However, the results of conventional visualization methods are difficult to locate in detail, the reason being that we are mainly visualizing high-dimensional features in the penultimate layer of the network and we use bilinear methods to restore extremely small pieces of information into their original size. Because our method generates an attention map from a segmentation map of the same size as the input image, it catches smaller infection regions and allows for a more detailed visualization.

## Methods

This study was approved by the Japan Medical Image Database (J-MID). All methods were performed in accordance with the guidelines and regulations of J-MID, and informed consent was obtained from all subjects and/or their legal guardian(s).

**Figure 1:**
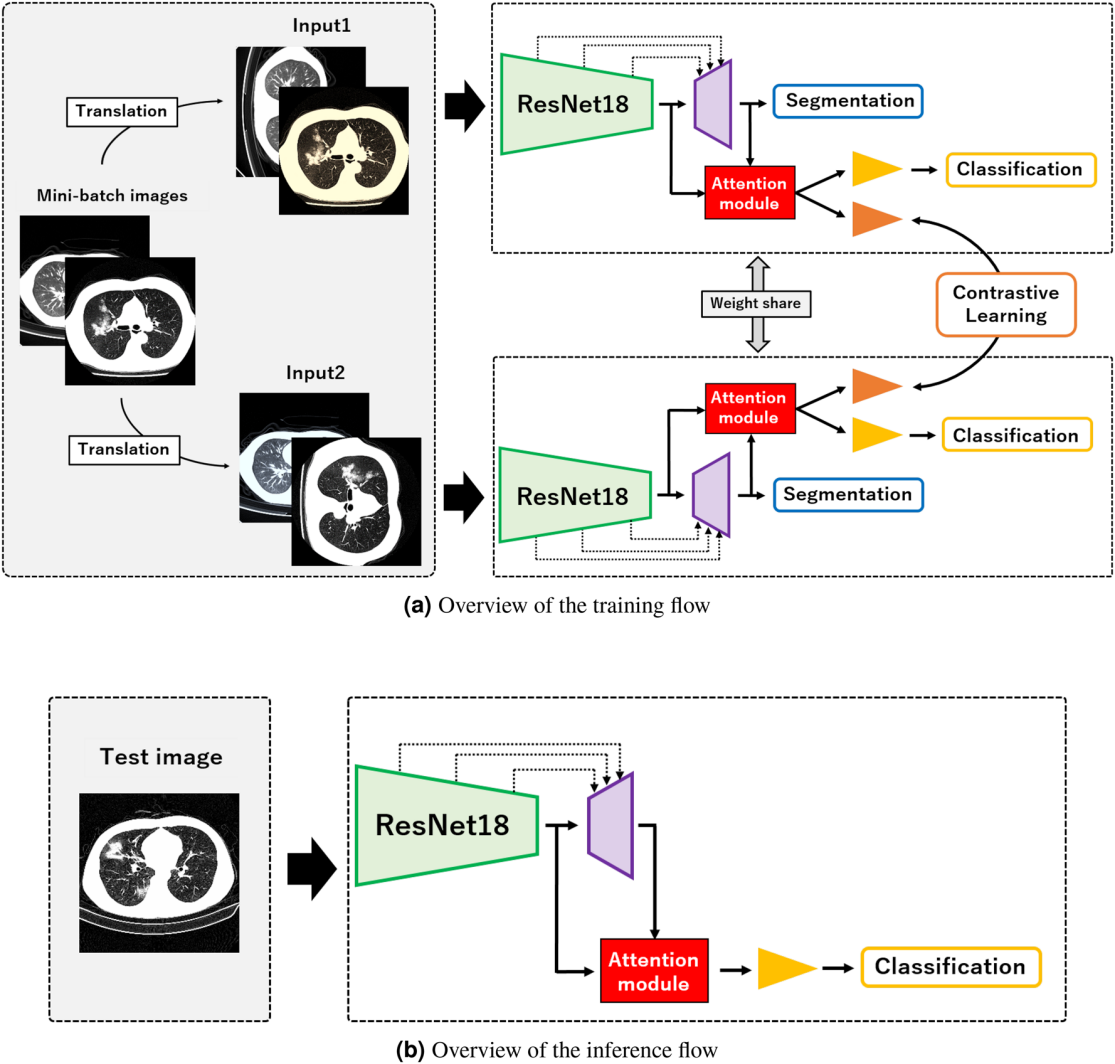
Overview of proposed method for the training and inference flows.

This section describes the overview of our method for classification and a visual explanation. Figure 1a shows an overview of the training flow, and Fig. 1b shows an overview of the inference flow of the proposed method. During training, two image pairs, which are affine and color transformed using the method described in^19^, are fed into the CNN, and high-dimensional features are obtained. The features are then fed into three networks, i.e., an FCN for classification, an FCN for contrastive learning, and a decoder for a semantic segmentation. The outputs of these networks are three types of vectors for classification, contrastive learning, and semantic segmentation. Herein, we describe the roles of three vector types: a vector of classification for classifying COVID-19 pneumonia, a vector of contrastive learning for creating a better feature space for classification, and a vector of semantic segmentation for classifying locations within the image at the pixel-level and leaking an attention location to the networks for classification and contrastive learning.

During an inference, test images are fed into the trained CNN, and we obtain only the classification result. We also visualize an important location related to classification from feature maps of the attention module. Unlike conventional contrastive learning^18–22^ and other visualization methods^23–28^, our proposed method does not require two-stage learning, and is able to generate a classification and visual explanation using only a single model.

**Figure 2:**
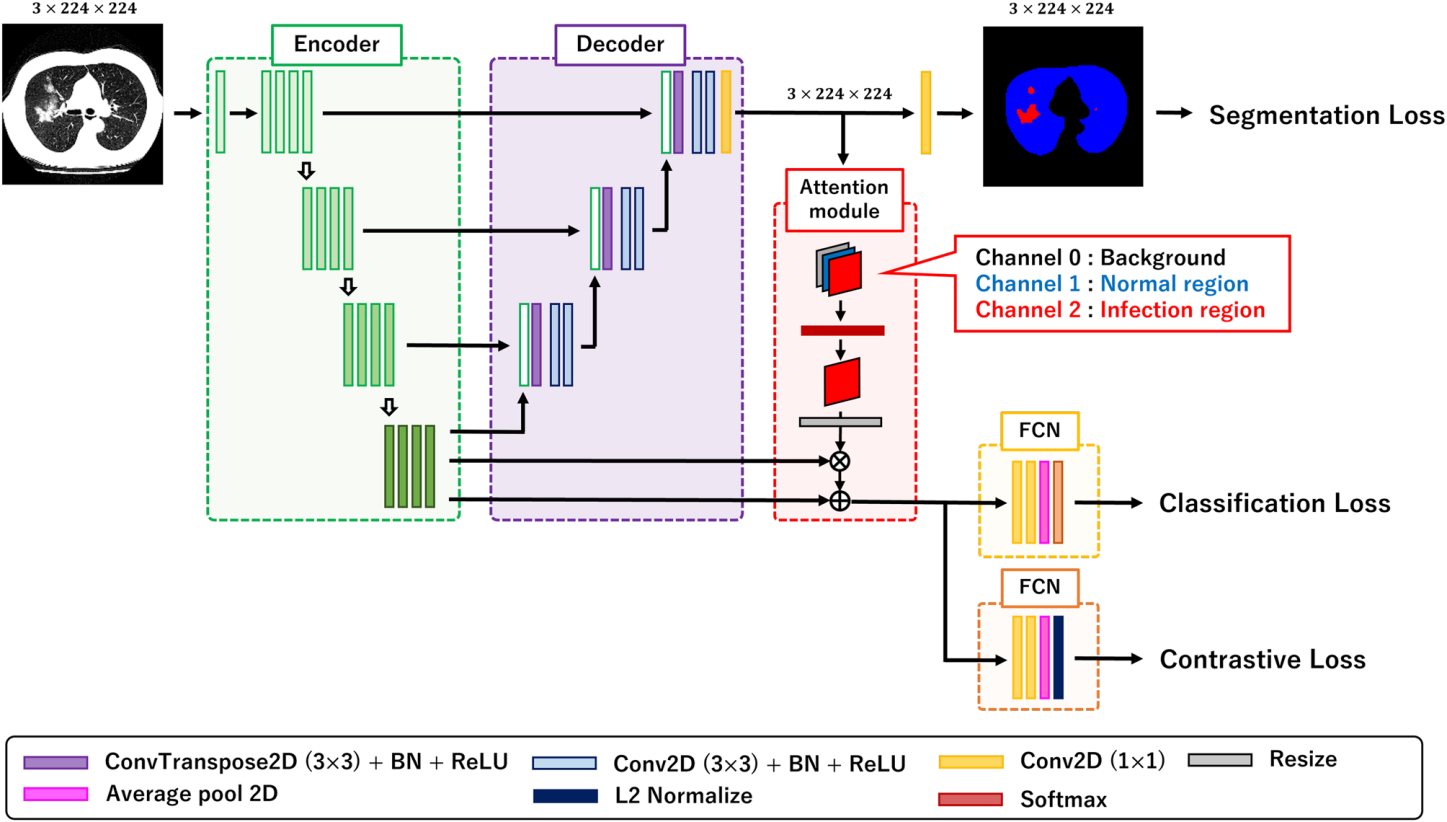
Overview of network structure. The proposed method is based on the U-Net architecture. The encoder consists of ResNet18, the output has high dimensional features, and the decoder outputs a segmentation map. The features extracted from ResNet18 are fed into two fully convolution networks (FCNs), and we obtain two types of vectors for classification and contrastive learning. The attention module also teaches the information of infection regions for two FCNs. A ground truth of a semantic segmentation includes three categories. A black region is a background category, and blue and red regions are normal and infection regions.

Figure 2 shows an overview of the network structure. The proposed network is has an encoder-decoder structure^15^, and the encoder network is a ResNet18 pre-trained using ImageNet^46^. The decoder network consists of a deconvolutional layer^47^, batch normalization^48^ and ReLU function, and outputs a segmentation result based on the point-wise convolutional layers along with the information from the encoder network. The features from ResNet18 are fed into classification and contrastive learning networks. These networks consist of two point-wise convolutional layers and a global average pooling layer^49^. In the classification network, the softmax function layer is used and the output is the probability of classification. In the contrastive learning network, an L2-Normalization layer is used and the network outputs 256-dimensional vectors for the cosine similarity.

The role of the attention module is for teaching an attention location to two networks for classification and contrastive learning. The feature map obtained from the decoder network has information on three categories in a CT-image: background, normal region, and infection region. The proposed attention module only retrieves the features of the infection region after the softmax layer and resizes the attention map to the size of the features from ResNet18. The feature maps are then multiplied by the attention map to generate a weighted feature map, and the weighted feature maps are added to the original feature maps.

During the experiments, we evaluated two types of methods. The proposed method using only classification and contrastive learning is called Double Net, and the method using a semantic segmentation and attention module is called Triple Net. Double Net is based on the hybrid network in^42^, and aims to confirm the effectiveness of the simultaneous learning of contrastive learning and classification. Triple Net aims to confirm the importance of teaching the attention location to the classifier. Although Triple Net needs both labels of classification and semantic segmentation, unlike conventional classification methods for COVID-19^4,50,51^, it can clearly visualize the location related to classification by doing segmentation simultaneously.

### Loss function

#### Classification loss

When there are *N* datasets $(\{ x_k,y_k \}_{k=1...N})$ of images $x_k$ and their labels $y_k$, because the datasets in the mini-batch include augmented images, the number of samples is 2*N* $(\{ \widehat{x_k},\widehat{y_k} \}_{k=1...2N})$. For classification of the loss function, we use the softmax cross entropy loss shown in Eq. (1), where *C* is the number of categories for classification, $t_{kc}$ is the teacher label, and $z_{kc}^{ce}$ is the predicted probability for class *k*. Because the softmax cross entropy loss is also applicable to the augmented images, it is applied to 2*N* samples in a mini-batch.

1 $$\begin{aligned} Loss_{ce} = -\sum _{k=1}^{2N} \sum _{c=1}^{C} t_{kc}\log z_{kc}^{ce} \end{aligned}$$

#### Contrastive loss

For contrastive learning, we adopted supervised contrastive learning^18^. The contrastive loss function is shown in Eqs. (2) and (3).

2 $$\begin{aligned} Loss_{cl}= & {} -\sum _{i=1}^{2N} \frac{1}{2N_{t_i}-1}(L_i^{cl}) \end{aligned}$$

3 $$\begin{aligned} L_i^{cl}= & {} \sum _{j=1}^{2N} {\mathbb {I}}_{i \ne j} {\mathbb {I}}_{t_i = t_j} log \frac{exp(z_i^{cl} \cdot z_j^{cl}/\tau )}{\sum _{k=1}^{2N} {\mathbb {I}}_{t_i \ne t_k} exp(z_i^{cl} \cdot z_k^{cl}/\tau )} \end{aligned}$$

In Eq. (3), *i* presents a sample from the true class, *j* presents samples having the same class as *i* (positive), and *k* presents samples having a different class from *i* (negative). In addition, ${\mathbb {I}}_{i \ne j}$ means that *j* is not the same image as *i*. Moreover, ${\mathbb {I}}_{t_i = t_j}$ also means that the teacher labels are of the same category, and ${\mathbb {I}}_{t_i \ne t_k}$ means that the teacher labels are of a different category. Therefore, Eq. (3) shows that all positive pairs contribute to the numerator, and all negative pairs contribute to the denominator for the features of the reference class of data in a mini-batch. Ideally, Eq. (3) should maximize the cosine similarity of the numerator and minimize the cosine similarity of the denominator, and we apply the training such that Eq. (3) is maximized. In fact, we minimize Eq. (2) with a negative sign to minimize the error using a gradient descent. Note that for each anchor i, there is 1 positive pair and $2N_{ti}-2$ negative pairs, and thus the denominator has a total of $2N_{ti}-1$ terms (positive and negative). Here, $\tau$ is a temperature parameter, and we use the same value as $\tau = 0.07$ from the original study^18^.

In the case of Double Net, the final loss function for classification and contrastive learning is described in Eq. (4). To control the balance of two-types training, we used a inversely proportional weighting coefficient $\lambda = 1 - epoch / epoch_{max}$ inspired by^42^, where *epoch* denotes the current epoch number and $epoch_{max}$ indicates the maximum epoch number. From the weighting, contrastive loss is prioritized during the early stage of training, and the model is trained using the ideal feature space. During the end of the training, the classification loss is prioritized, and the model is trained to obtain a more accurate prediction. Conventional classification methods using contrastive learning^18–22^ apply contrastive learning during the first step, and then train only a new classifier by fixing the weights of the network at the first step. The proposed weighting schedule aims to realize a one-stage learning method applied in two steps.

4 $$\begin{aligned} Loss_{double} = \lambda \cdot Loss_{cl} + (1 - \lambda ) \cdot Loss_{ce} \end{aligned}$$

#### Segmentation loss

For semantic segmentation loss, we adopted the Dice loss^16^ in Eq. (5), where *C* is the number of categories for segmentation, *n* is the number of pixels, $z_{nc}^{seg}$ is a predicted segmentation, and $z_{nc}^{seg'}$ is an annotation of semantic segmentation. Here, $\gamma$ is added to both the numerator and denominator to ensure that the function is not undefined in edge case scenarios, such as when $z_{nc}^{seg} = z_{nc}^{seg'} = 0$, and we set $\gamma = 1$. In the case of Triple Net, a final loss function for the three types of learning is as shown in Eq. (6).

5 $$\begin{aligned} Loss_{seg}= & {} \frac{1}{C}\sum _{c=1}^{C} \left( 1 - \frac{\sum _{n} z_{nc}^{seg} z_{nc}^{seg'} + \gamma }{\sum _{n} (z_{nc}^{seg})^2 + \sum _{n} (z_{nc}^{seg'})^2 + \gamma }\right) \end{aligned}$$

6 $$\begin{aligned} Loss_{triple}= & {} \lambda \cdot Loss_{cl} + (1 - \lambda ) \cdot Loss_{ce} + Loss_{seg} \end{aligned}$$

## Experiments

### Datasets and training conditions

#### Dataset

As the dataset, we used the CT volumes taken in multiple medical institutions in Japan. We used CT volumes of all 1,279 patients registered in the J-MID database, and there are CT scans with annotation and CT slices for classification and semantic segmentation. The specifications of the CT volumes are as follows: a 16-bit pixel resolution of $512 \times 512$, 56 to 722 slices, a pixel spacing of 0.63 to 0.78 mm, and a slice thickness of 1.00 to 5.00 mm. The ground truth for COVID-19 pneumonia was checked by radiologists of the “Japan Radiological Society” based on^1^, and that for semantic segmentation was created by medical image processing researchers and checked by doctors^17^. The ground truth for pneumonia were classified into four types of image findings in^1^: a typical appearance, an indeterminate appearance, an atypical appearance, and a negative outcome for pneumonia. Ground truth images for segmentation contain three categories, i.e., the background, normal regions, and infection regions. Some of the image slices in a CT volume do not sufficiently show the lung area. In addition, the number of slices is not uniform among the samples, and thus it is difficult to use them as input. We therefore either selected a single CT image having the largest infection region or an image having the largest normal region from the segmentation results. We also used a gray-scale of $-1000$ to $-500$ within the 16-bit images, converting them from 16-bits into 8-bits and resizing them to a pixel resolution of $256 \times 256$ for easier handling.

We evaluated the binary classification and four-class classification on these datasets. The details of the dataset are shown in Table 1. we used 470 samples as the typical appearance, 289 samples as the indeterminate appearance, 137 samples as the atypical appearance, and 383 samples as the negative outcome for pneumonia. For binary classification, the categories of both the typical appearance and the indeterminate appearance were treated as a single category (positive), and the categories of both atypical appearance and negative outcome for pneumonia were treated as another category (negative). We used 759 samples as the positive category and 520 samples as the negative category. We divided each dataset into 2 to 1 in numerical order, and made them for training data and for inference data. In inference data, we also divided it into 1 to 2 for validation data and for test data. For example, the first time of cross validation for binary classification, we used 853 samples for training data, 138 samples for validation data and 288 samples for test data. Our experiments were conducted based on a three-fold cross validation while switching training data and inference data that were divided 2 to 1, and we evaluated the accuracy using only test data in inference data.

**Table 1:** Datasets used for evaluation.

Dataset	Binary classification		Four-class classification			
	Positive	Negative	Typical appearance	Indeterminate appearance	Atypical appearance	Negative for pneumonia
The number of samples	759	520	470	289	137	383

#### Training conditions

The batch size was set to 32, the number of epochs was set to 1000, and the optimizer was Adam^53^ with a learning rate of 0.001. For data augmentation, we applied several random on-the-fly data augmentation strategies during training, including images randomly cropped to $224\times 224$, rotated with an angle randomly selected within $\theta = -90$ to 90, flipped horizontally, and having random changes in the brightness values. For data pre-processing, we applied a normalization of 0 to 1 and subtracted the per-pixel mean^15^. Experiments were conducted based on a three-fold cross validation, and the average accuracy of three experiments was used for the final evaluation. In all experiments, we set random seed to zero.

For compared methods, we used the standard ResNet18 pre-trained on ImageNet^46^ (Baseline), weakly supervised deep learning (WSDL)^4^, an attention branch network (ABN)^44^, and multi-task deep learning (MTDL)^7^ as comparison methods. WSDL and MTDL are methods for COVID-19 infection classification using CT-images. An ABN is a method for achieving a visual explanation using an attention mechanism. The bold letters present the best accuracy in the tables. Furthermore, we evaluated that the encoder of Triple Net based on ResNet18 to the network used by WSDL (Triplet Net + WSDL). WSDL can handle the features of various resolutions, and we consider that the encoder with WSDL can outperform other comparison methods due to the features based on infection regions of different sizes. In addition, we also compared 3D networks^50–52^ using dataset consisted of CT volumes to confirm the difference in performance between 2D CNN and 3D CNN. In this study, we set the frame size to 64.

For the evaluation metric, we used the accuracy, precision, sensitivity, and specificity for binary classification and four-class classification as following^4,7^. We also used F-measure to evaluate the fairness of predictions. Furthermore, we carried out the analysis of the area under the receiver operating characteristic curve (AUC) for a quantification of our classification performance for a binary classification as following^4,7^.

### Results

#### Learning on binary classification

Table 2 presents the evaluation results of test images for binary classification. In Table 2, the accuracy was improved by over 1.74% when we used Double Net, and over 4.87% when we used Triple Net, in comparison with the baseline. Similarly, in comparison with the baseline, the precision was improved by 1.09%, the sensitivity by 9.04%, the specificity by 2.12%, the F-measure by 4.69% and the AUC by 2.09%. Furthermore, the accuracy using Triple Net + WSDL was higher than that using only Triple Net. The F-measure was improved by 1.83 % and the AUC was improved by 0.94 % in comparison with only Triple Net. We confirmed the effectiveness of teaching an inflamed area to the classifier, and compared to conventional methods, our proposed methods achieved the highest accuracy under all evaluation measures. Adding contrastive learning and an attention mechanism was effective in comparison with the conventional methods for COVID-19 infection classification. On the other hand, 3D-ResNet18 has the worst accuracy compared to other methods. We consider that the difference in accuracy between 2D CNN and 3D CNN is due to the usage of pre-trained model. Although our 2D CNN models like ResNet18 are pre-trained on the ImageNet dataset, pre-trained 3D CNN models are only for the action recognition task^55^ and they are not suitable for medical image dataset.

**Table 2:** Comparison results for binary classification.

Tasks	Accuracy (%)	Precision (%)	Sensitivity (%)	Specificity (%)	F-measure (%)	AUC (%)
**3D network**						
3D-ResNet18^52^	71.02_±1.25_	50.35_±6.68_	71.34_±4.78_	71.36_±1.94_	58.52_±3.16_	76.56_±2.21_
CovNet (ResNet18)^50^	74.39_±1.65_	56.33_±13.70_	76.72_±5.52_	74.82_±4.94_	63.41_±6.88_	84.36_±0.61_
DeCovNet^51^	73.58_±1.07_	63.98_±5.53_	69.35_±1.67_	76.36_±2.44_	66.38_±2.58_	80.50_±1.32_
**2D network**						
Baseline (ResNet18)	73.59_±2.88_	65.67_±3.87_	68.55_±3.35_	76.84_±2.64_	67.07_±3.58_	80.79_±2.06_
WSDL^4^	75.56_±1.38_	62.82_±7.31_	74.50_±5.34_	76.79_±2.79_	67.63_±2.73_	83.25_±1.90_
ABN^44^	76.03_±3.44_	62.89_±10.09_	74.51_±1.83_	77.09_±4.57_	67.83_±6.75_	83.59_±3.77_
MTDL^7^	75.79_±2.08_	61.96_±3.89_	74.98_±4.28_	76.41_±1.82_	67.71_±2.59_	81.04_±4.39_
Double Net (ours)	75.33_±1.33_	**70.70**_±**4.36**_	70.01_±3.76_	79.53_±1.20_	70.12_±0.57_	80.65_±0.79_
Triple Net (ours)	78.46_±0.40_	66.76_±0.67_	**77.59**_±**1.28**_	78.96_±0.20_	71.76_±0.16_	83.68_±1.90_
Triple Net + WSDL^4^ (ours)	**79.40**_±**2.71**_	70.15_±4.47_	77.53_±4.00_	**80.60**_±**2.42**_	**73.59**_±**3.65**_	**84.62**_±**2.77**_

**Figure 3:**
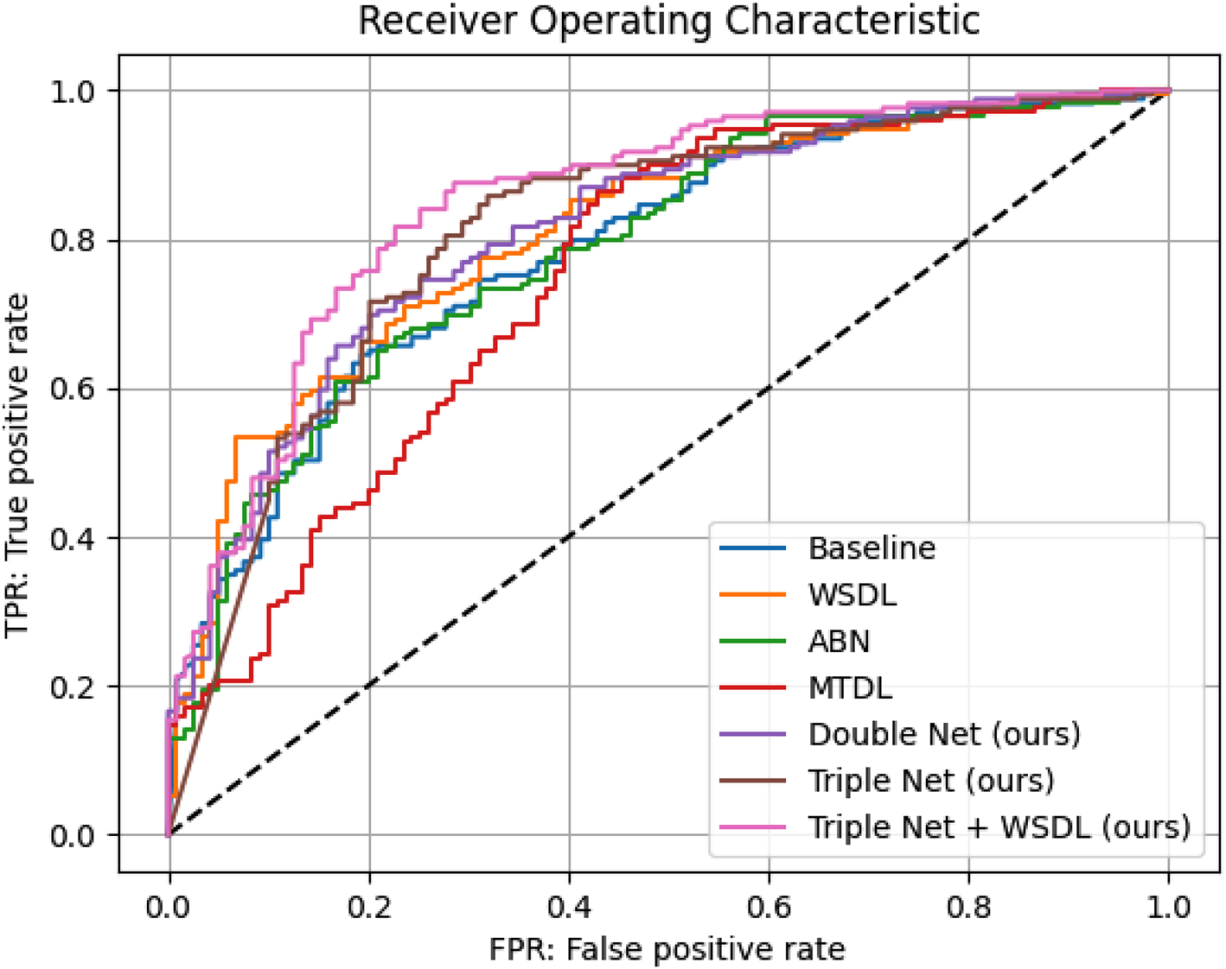
Receiver operating characteristic (ROC) of various methods for binary classification.

Figure 3 presents the receiver operating characteristic (ROC) of various methods for binary classification. Our proposed methods are shown in the purple, brown and pink graphs. In Fig. 3, the graph of Triple Net + WSDL was closest to the upper left, demonstrating that it achieved the highest performance. In fact, the AUC of Triple Net showed the highest accuracy in comparison with the other methods.

**Figure 4:**
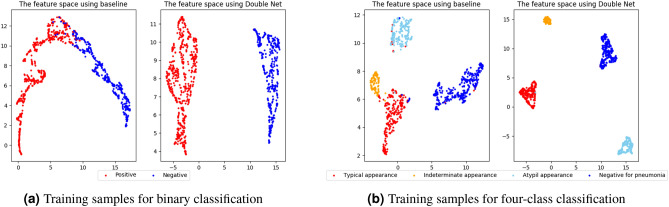
Visualization results of features at last convolutional layer of ResNet18 when we used the training samples. We compressed the features into two dimensions using UMAP^54^ for (**a**) binary classification and (**b**) four-class classification.

Figure 4a presents the visualization results of the features at the last convolutional layer of ResNet18. We compressed the features into two dimensions using UMAP^54^. The column on the left shows the results of the baseline and the column on the right shows the result of Double Net. The red dot indicates a positive category, and a blue dot represents a negative category. For the baseline, although most of the samples were separated between categories, there were points where the features of other categories overlap near the center. However, as shown in Double Net, each category was the independent, and it was possible to create the feature space for separating all categories. Because this feature space was separated into two categories, the network prediction based on the separated features prevented an incorrect prediction.

#### Learning on four-class classification

Table 3 shows the performance for four-class classification. As presented in Table 3, our Double Net and Triple Net were better performance than the baseline, and improved the accuracy by 1.63% and 4.54%. Furthermore, Triple Net + WSDL achieved the best performance in comparison with conventional methods. In comparison with the baseline, it was improved the accuracy by 8.47%, the precision by 5.17%, the sensitivity by 7.71%, the specificity by 9.22% and the F-measure by 4.48%. WSDL uses the features of both the upper and lower layers, and we consider that the features of the upper layers with finer information are required for classification of the classes with large area in four-class classification. Actually, Triple Net + WSDL improved the F-measure and sensitivity metrics by 3.21% and 2.67% in comparison with the original WSDL. We confirmed that our proposed methods using contrastive learning and an attention module were effective even if the number of classes increased.

**Table 3:** Comparison results for four-class classification

Tasks	Accuracy (%)	Precision (%)	Sensitivity (%)	Specificity (%)	F-measure (%)
**3D network**					
3D-ResNet18^52^	45.39_±3.16_	36.91_±2.58_	36.82_±3.65_	44.84_±2.97_	35.06_±2.73_
CovNet (ResNet18)^50^	56.81_±0.23_	45.78_±2.20_	43.26_±5.53_	51.61_±6.25_	41.51_±4.25_
DeCovNet^51^	50.63_±3.79_	45.42_±2.69_	45.30_±2.11_	49.99_±3.65_	44.58_±2.56_
**2D network**					
Baseline (ResNet18)	49.48_±2.50_	42.05_±1.34_	41.11_±1.71_	48.67_±2.54_	40.82_±1.48_
WSDL^4^	53.66_±1.48_	44.04_±0.80_	45.61_±2.80_	53.13_±1.37_	42.63_±0.84_
ABN^44^	51.10_±0.67_	42.26_±0.96_	41.81_±1.62_	50.34_±0.65_	41.32_±1.07_
MTDL^7^	52.38_±3.36_	42.31_±2.39_	40.49_±3.83_	47.38_±6.95_	40.42_±2.96_
Double Net (ours)	51.11_±1.52_	41.23_±1.63_	39.62_±1.75_	50.35_±1.61_	39.87_±1.65_
Triple Net (ours)	54.02_±2.30_	43.84_±2.63_	40.37_±4.56_	44.51_±6.40_	41.70_±3.71_
Triple Net + WSDL^4^(ours)	**58.22**_±**3.35**_	**47.22**_±**3.06**_	**48.82**_±**4.69**_	**57.89**_±**3.31**_	**45.30**_±**3.65**_

Figure 4b shows the visualization results of features compressed similarly to a binary classification. The left column presents the result of the baseline, and the right column shows the result of Double Net for four-class classification. Red dots indicate a typical appearance, orange dots shown an indeterminate appearance, aqua blue dots illustrate an atypical appearance, and blue dots represent a negative outcome. In the case of the baseline, although each category was independent, there were some dots in which the distance between categories was close, and dots that were close to different category sets. Such results are caused by a misclassification. However, in the case of Double Net, the distance between all categories was sufficiently large. These results demonstrate the effectiveness of contrastive learning, which creates a space in which images within the same categories are closer together and images of different categories are kept at a distance, even if the number of classes increases.

**Figure 5:**
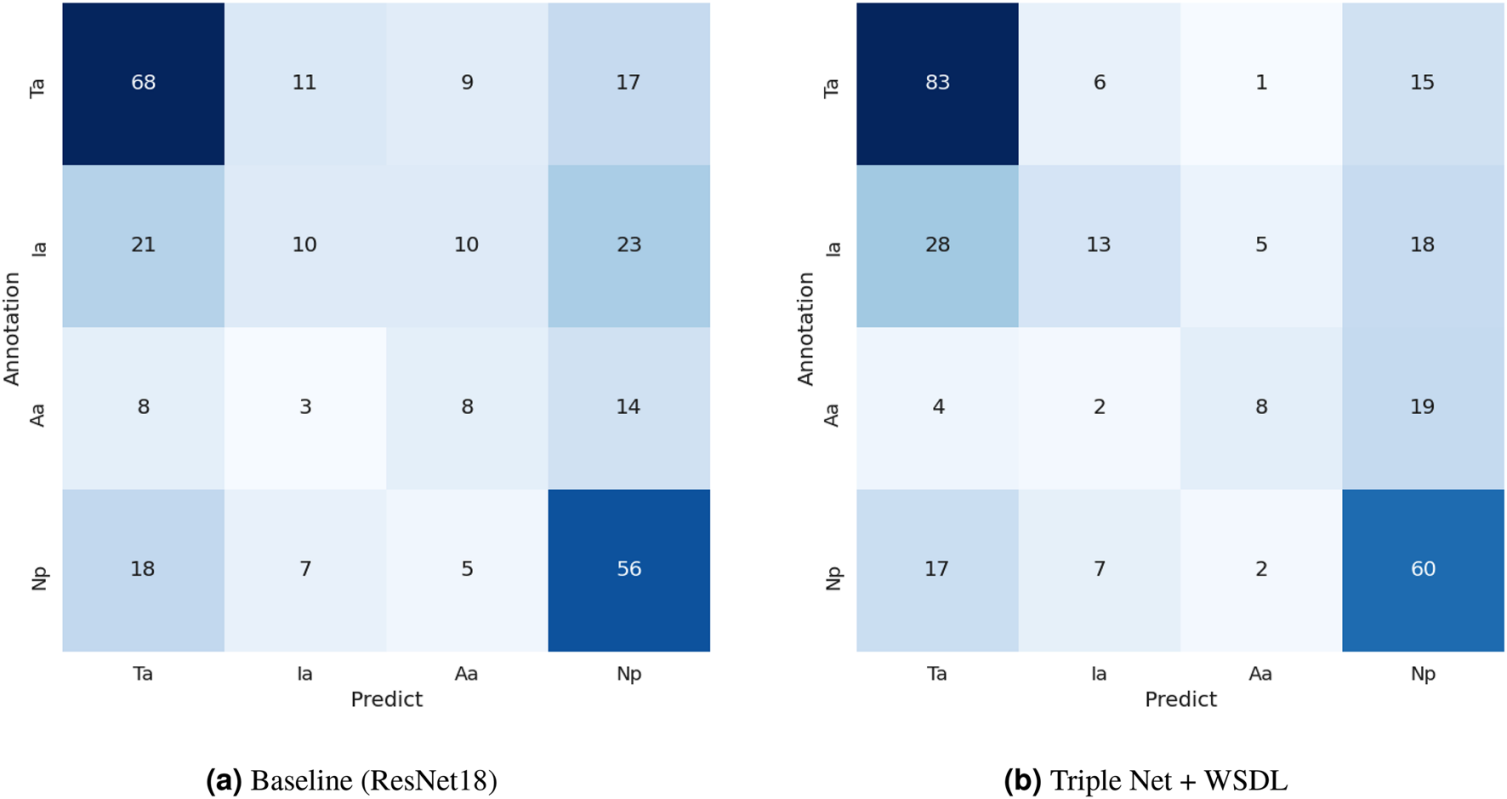
Evaluation results with confusion matrix using four-class classification. The left column presents the result of the baseline, while the right column shows the result of the Triple Net + WSDL. In the confusion matrix, *Ta* is the typical appearance, *Ia* is the indeterminate appearance, *Aa* is the atypical appearance and *Np* is the negative for pneumonia category.

Figure 5 shows evaluation results with confusion matrix using four-class classification. Especially, the number of correct for typical appearance category was increased, and the number of misclassification including positive categories was decreased. Although the number of correct for the atypical appearance was the same, it was often mistaken as the negative category for pneumonia, and it was reduced the mistakes as positive categories (the typical appearance and the indeterminate appearance). We consider that these results demonstrate the effectiveness of our proposed contrastive learning considering the relationships between classes and attention mechanism getting infection regions.

#### Results of visual explanation

**Figure 6:**
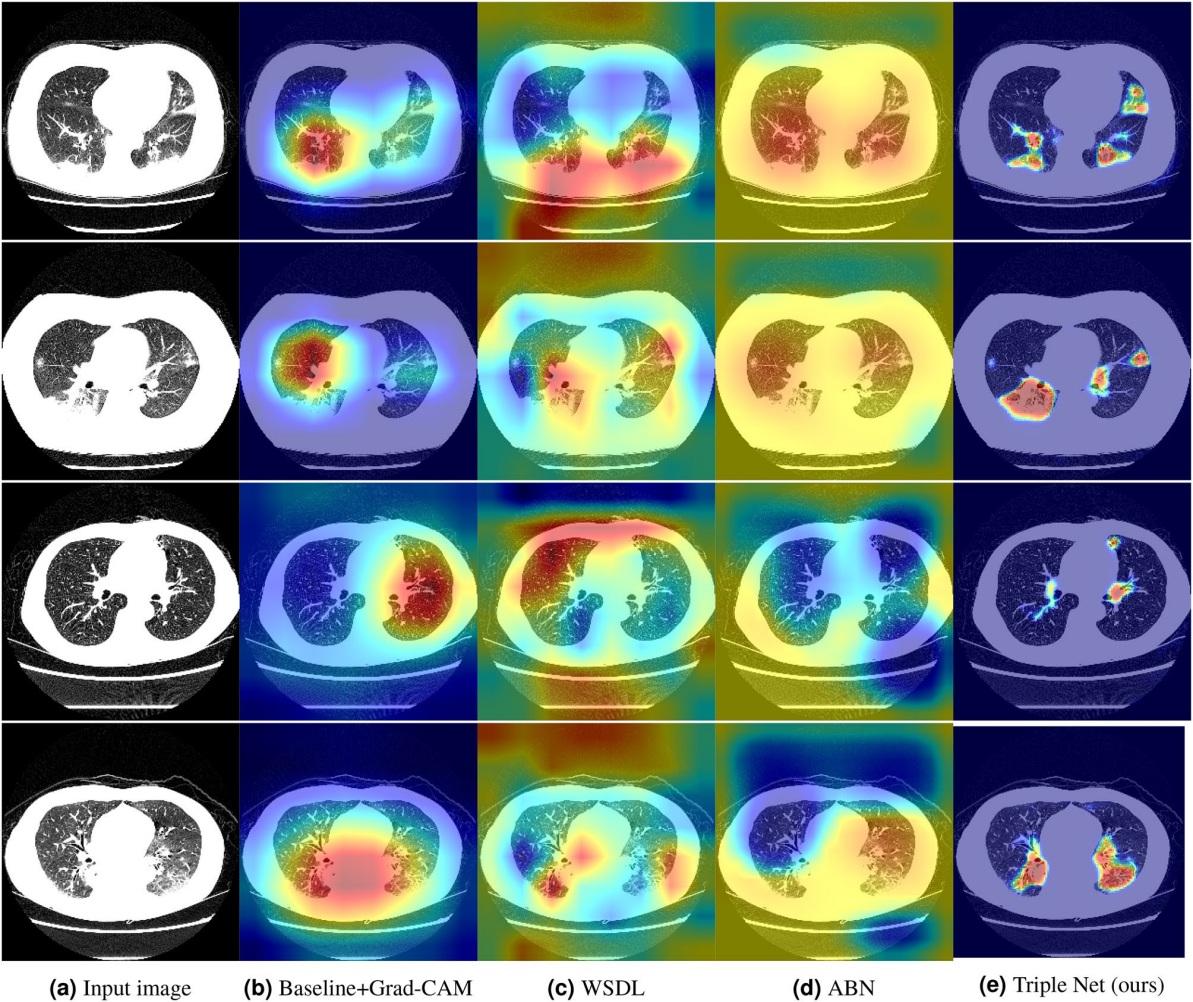
Results of visual explanation. (**a**) Input image, (**b**) baseline with Grad-CAM^23^, (**c**) WSDL with CAM^4^, (**d**) attention map using an ABN^44^, and (**e**) the attention map achieved by Triple Net (ours). All explanation images are a superposition of input images and heat maps. Red shows the most important location, and blue indicates an unimportant location for classification.

Figure 6 shows the results of the important location for a binary classification. The first and second rows are visualizations of positive categories, and the third and fourth rows are visualizations of negative categories, under the condition in which a prediction is correct. Red shows the most important location, and blue shows an unimportant location for classification. We compared Triplet with the baseline, WSDL, and ABN. The baseline was visualized using Grad-CAM, WSDL was visualized using the CAM, and both the ABN and Triple Net were visualized using an attention map. In the case of the baseline with Grad-CAM, the area in the lung field was reddish. However, the heat map was blurred, and it was difficult to recognize the inflammation in detail. In the case of WSDL and the ABN, there were many responses outside of the lung areas, and the results were poor for making a proper judgment. In the case of Triplet Net, it was possible to visualize the detailed basis of the decision making by specifying more finely within the lung field region in comparison with the conventional method. Although our visualization method has to prepare segmentation labels, comparison visualization methods without segmentation label cannot get infection regions precisely. It is too ambiguous to understand the judgement reason for human because the heatmap generated by Grad-CAM reacts to regions except for the lung area. From these results, we confirmed that the proposed attention mechanism visualized using features of segmentation a better understanding for human viewers.

**Figure 7:**
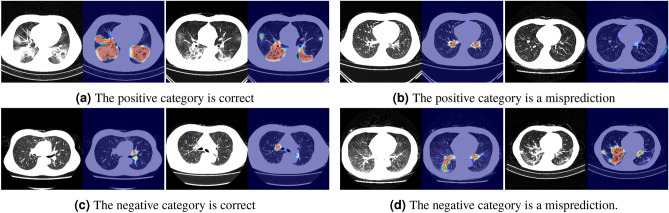
Results of visual explanations for binary classification. (**a**,**c**) Results when predictions are correct. (**b**,**d**) Results when predictions are wrong.

Figure 7 shows the visualization results when our method misclassified the binary classification. When the predictions were correct, as shown in Fig. 6a and c, the sample in the positive category emphasized the infection areas in the lower area of the lung field. In the negative category, we confirmed that the heat map was made by looking at the blood vessels. When the predictions were incorrect, as shown in Fig. 6b and d, the attention map did not respond to inflammatory areas in the positive category, and the negative categories were often mistaken for the lung areas unrelated to inflammatory regions such as blood vessels.

**Figure 8:**
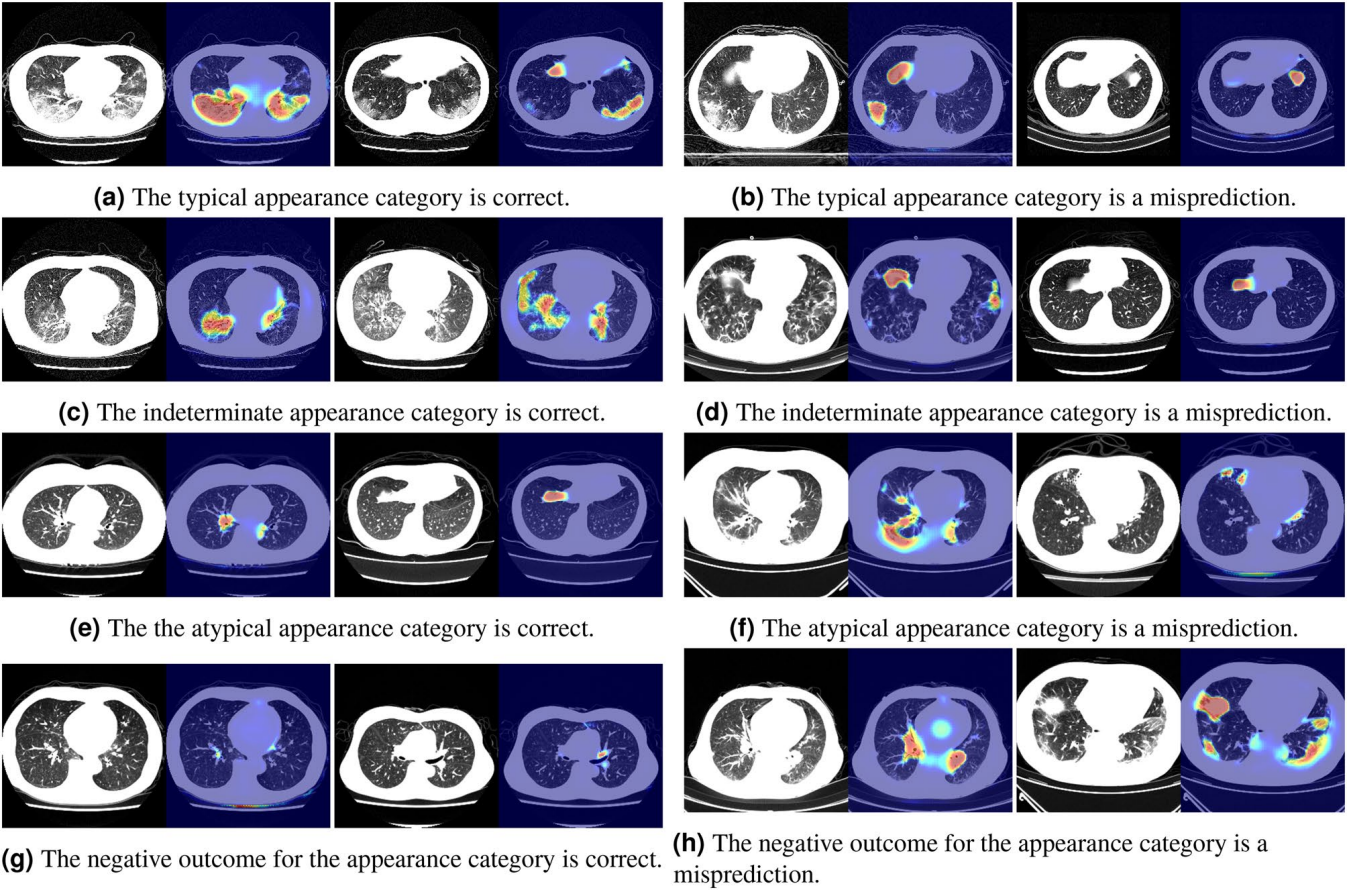
Results of visual explanations for four-class classification. (**a**,**c**,**e**,**g**) Results when the predictions are correct. (**b**,**d**,**f**,**h**) Results when the predictions are incorrect.

Figure 8 shows the visualization results when our method misclassified the four-class classification. In Fig. 8a and c, when the prediction was correct, the samples in the typical appearance category emphasized the infection areas in the lower lung fields, and the samples in the indeterminate appearance category emphasized the intermediate infection areas. In the categories of an atypical appearance and a negative outcome, there were very little reactions from the large heat map (Fig. 8e and g). When the predictions were incorrect, the attention map did not respond to inflammatory areas in either the typical or indeterminate appearance (Fig. 8b and d). In addition, the atypical appearance were often mistaken in the samples of ambiguous inflammatory areas (Fig. 8f), and the negative outcome were mistaken in the lung areas unrelated to inflammatory areas (Fig. 8h). Then, by checking the 3D lung regions in Fig. 8f and h, we confirmed that the samples where the inflammatory areas extending to the slice images were mistaken for the typical appearance category, and the samples where no inflammatory areas were mistaken for the negative outcome for the appearance category in many cases in Fig. 8f. In the case of Fig. 8h, although there were also no infection regions in the other slice images, the pleural effusion regions were often mistakenly classified as the infection. These visualizations demonstrate that the model predicted the result based on the infection area.

#### Discussion

The limitation of our proposed method is to require an infection segmentation mask in training. Although conventional classification methods using CT volumes^50,51^ compared by this study do not require an infection segmentation label and input CT volumes directly into the model, an input of our proposed Triple Net is used a slice image with the largest infection region from CT scan by infection segmentation masks.

However, as shown in Tables 2 and 3, Triple Net was the best accuracy in comparison with the methods without the infection segmentation labels^4,50,51^. From those decisive results, we consider that the information of input is missing because the CT volumes consisting of different number of slices are aligned to have the same number of slices to be handled when we used 3D volumes as inputs. Then, it is considered that the slice selection using the infection segmentation mask can make a better decision using the infection regions.

Furthermore, as show in Fig. 6, Triple Net was possible to visualize the detailed basis of the decision making in comparison with the Grad-CAM and WSDL, and we consider it is important that teaching infection regions directly to the deep neural network using segmentation mask. Therefore, although there is a limitation to use the segmentation mask, it is important to use the segmentation mask from the viewpoint of classification and visualization in the case of COVID-19 from CT images.

## Conclusion

In this study, we designed a novel classification method for COVID-19 infection from CT-images. In the F-measure, our Triple Net + WSDL achieved about 73.59% in binary classification and about 45.30% in four-class classification. Furthermore, we confirmed that proposed contrastive learning generated a better feature space even when the dataset included images taken with various shooting equipment, and the attention module contributed to the specifics of the infection areas. However, the accuracy of the four-class classification may be further improved, which will be achieved by including more accurate information on the four classes of the inflammatory regions. This remains an area of future research.

## Data Availability

The data that support the findings of this study are available from J-MID, but restrictions apply to the availability of these data, which were used under license for the current study, and so are not publicly available. Data are however available from the authors when you become a member of J-MID (http://www.radiology.jp/j-mid/).
